# Mindmelding: Connected Brains and the Problem of Consciousness

**DOI:** 10.4103/0973-1229.38516

**Published:** 2008

**Authors:** William Hirstein

**Affiliations:** * *Chair, Department of Philosophy, Elmhurst College, Elmhurst, I llinois, USA*

**Keywords:** *Consciousness*, *Executive processes*, *Association fibers*, *Binding*, *Prefrontal Cortex*, *Self*, *Subjectivity*

## Abstract

Contrary to the widely-held view that our conscious states are necessarily private (in that only one person can ever experience them directly), in this paper I argue that it is possible for a person to directly experience the conscious states of another. This possibility removes an obstacle to thinking of conscious states as physical, since their apparent privacy makes them different from all other physical states. A separation can be made in the brain between our conscious mental representations and the other executive processes that manipulate them and are guided by them in planning and executing behaviour. I argue here that these executive processes are also largely responsible for producing our sense of self in the moment. Our conscious perceptual representations themselves reside primarily in the posterior portions of the brain's cortex, in the temporal and parietal lobes, while the executive processes reside primarily in the prefrontal lobes. We can imagine an experiment in which we sever the association fibers that connect the posterior regions with these prefrontal regions and, instead, connect the posterior regions to the prefrontal regions of another person. According to my hypothesis, this would produce in the latter person the direct experience of the conscious perceptual states of the first person.

## Introduction

Working inward from the sense organs, neuroscientists have begun to isolate those brain areas and processes important for consciousness. These are good days for mind-body materialists, who have held all along that the mind could be understood in sheerly physical terms. Yet a final, troubling impasse remains. All of our current research techniques leave the scientific observer of the brain locked out of the experience the subject herself is having. Using their new imaging technologies, scientists can observe all sorts of brain activity, but it seems they can never detect the most crucial properties of conscious states, the ones the subject herself is aware of. If someone is looking at a blue sky, for instance, the scientists monitoring her brain cannot detect anything blue. This describes the current state of research, but will the scientists of the future ever enter that sacred citadel, the mind itself? If they cannot, if conscious states are necessarily and permanently private, this creates a fundamental problem for materialism. If mental states lack a basic feature possessed by all other known physical states - the capacity to be observed or experienced by many people in many ways - this gives us firm reason to suspect that they are not truly physical in any way we now understand that concept. Dualists still have good reason to cling to the possibility that the mental and the physical are disjoint categories. The following argument against materialism seems as strong as ever:

## The Privacy Argument

**Premise 1:** No physical states are private.

**Premise 2:** All conscious states are private.

**Conclusion:** No conscious states are physical states.

This piece of reasoning causes those materialists who approach it to scatter, giving rise to several different schools of thought as to how to defuse it. As phrased above, the argument is formally valid, which implies that anyone who disagrees with it cannot dispute that the conclusion follows logically from the premises. This means that any materialist who disputes the conclusion must find something wrong with one or both of the premises. There are three responses currently being pursued by three different groups of materialists:

### First Response: The First Premise is False

One group argues that there are brain states that can only be directly experienced by one person but are, nevertheless, physical states. This has led them to posit the existence of a previously unknown category of entities, or what philosophers call a *metaphysical category*. According to John Searle, their most prominent member, there is a special category of private physical states that includes our conscious states (Searle, 1994). One dramatic consequence of this view is that conscious states are different from other physical states in that a separation cannot be made between their existence and their owners’ knowledge of them. In philosophical terms, their *ontology* is necessarily bound to their *epistemology*. For them, to be is to be perceived; to exist is to be an object of someone's awareness.

Almost all of today's writers on consciousness accept this privacy - or what some refer to as the *subjectivity* - of mental states. Examples of this go back at least as far as John Locke, who said that we can never truly know what is in the mind of another, “because one man's mind could not pass into another man's body to perceive what appearances were produced” (Locke, 1690). Among contemporary writers, materialists such as Searle and dualists such as Thomas Nagel agree on privacy. Searle says that, “though I can easily observe another person, I cannot observe his or her subjectivity” (Searle, 1992). According to Nagel, “every subjective phenomenon is essentially connected with a single point of view, and it seems inevitable that an objective, physical theory will abandon that point of view” (Nagel, 1974). From this he concludes that, “the subjectivity of consciousness is an irreducible feature of reality” (Nagel, 1986).

A response that is frequently made to this approach is to reach for something known as *Ockham's razor*, an ancient maxim of metaphysics. “Entities should not be multiplied beyond necessity,” said William of Ockham, a medieval metaphysician; that is, do not take the extreme measure of positing new categories unless you are certain that an explanation cannot be constructed using the existing categories. There is always room for the revisionists to argue, however, that in this case the new category of entities is necessary in order to explain the mind. Surely though, there is force to what Ockham said: Given that the world is hard enough to understand, there needs to be some very clear positive reason for creating new categories of things or properties. It should not be something that we find ourselves backed into merely because we cannot figure something out. Are we satisfied that we have exhausted every possible way to successfully conceptualize conscious states as straightforward physical states?

### Second Response: The Second Premise is False

Perhaps good communication is the way around the wall of privacy. According to Daniel Dennett, a combination of investigation from the outside and verbal reports of subjects is sufficient for us to gain all the knowledge we need about conscious states, so that there is nothing significant left over that is truly private. We can interview subjects at great length, posing further questions to them to make sure we have understood what they are saying (Dennett, 1991). But despite our talent for communication, there is a huge difference between actually having these experiences and hearing verbal reports about them. Anyone who has ever tried to describe a dream to someone can attest to this. Verbal reports, no matter how thorough, can still be inadequate, misleading, or simply misinterpreted. The person hearing the verbal report is even farther away from the conscious state than the scientist observing the functioning brain. Dennett's notion that the richness and subtlety of conscious experience can be captured in language also strikes one as deflationary. Verbal communication may work for thoughts that occur in linguistic form, but much of our mental lives consist of images and emotions.

### Third Response: We Will Never Know Whether the Premises are True or False

Others have suggested that we humans have simply met our intellectual match – a problem we cannot and never will solve. According to a group that has come to be called the *mysterians*, the problem of relating consciousness to the physical realm is fated to remain a mystery. Colin McGinn, their primary spokesman, provides an interesting argument by analogy: Humans are biological organisms, formed by the processes of evolution. Other such creatures have limits to their mental capacities that are quite evident to us. Dogs can never understand calculus, for instance. Why should we not think that we too have limits? Not surprisingly, McGinn is especially daunted by the wall of privacy: “If your friend is staring at something green,” he says, “you cannot look at her and see the ‘greeniness’ of her experience. Such intimacy is ruled out by the nature of consciousness” (McGinn, 1999). “This is not just an accidental fact;” according to McGinn; “consciousness is *necessarily* not perceptible” (McGinn, 1999). Not only is consciousness essentially private in its natural state, but there is no possible way to extend our knowledge to breach the wall of privacy; no way we could change ourselves; no instrument we could invent that would be of any use. As he says:

There is no way to modify or extend introspection and perception so that they can transcend their present limitations. That is like hoping that if we tinker with our sense of touch it will eventually give us perceptions of color (McGinn, 1999). We cannot even conceive of a type of sense organ that would enable us to perceive consciousness (McGinn, 1999).

It is disappointing that something so close to us should prove permanently unfathomable. Normally, when one thinks of the limits of human knowledge, one thinks of distant events in space or time. Will we ever know whether there is life on other planets? Will we ever understand how the universe began? Given that neuroscientists are progressing rapidly and only just beginning to understand the higher levels of brain function, it seems absurd to give up now. If at some point well in the future, when neuroscientists are satisfied that they fully understand the brain, they still cannot find any coherent way to describe conscious states in straightforward physical terms, then perhaps we should worry.

## An Alternative View

I too am going to argue that the second premise is false, but not in the deflationary way that Dennett does. Dennett lets us know about another person's conscious states by limiting what there is to know, not by expanding our abilities to know. Contrary to this, I think that our knowledge can be expanded in this realm. The way around the impasse is to question the cherished and little-examined assumption that one person can never directly experience the mind of another. We are beginning to understand where in the brain the different constituents of conscious states reside, as well as how the brain knits them together to form the unified, coherent mental events we experience. I believe that this opens up the possibility of theorizing about how we might connect two brains to allow this sort of knowledge; to allow for the sharing of conscious states (Hirstein, 2001).

The possibility of one being having direct knowledge of the consciousness of another is already familiar to us, of course. Many of us were brought up with the belief that God knows everything that happens in our minds. There are also science fiction tales about this happening, from the “mindmelding” of the television series *Star Trek*, where Mr. Spock merges his consciousness with his subject, to the movie *Strange Days*, which depicts a device that can record one person's conscious experiences and allow another person to re-experience them by putting on a special headset and playing the recording. The movie *Being John Malkovich* also depicts interesting cases in which one person experiences the consciousness of another. It seems at least *conceivable* that I could experience your consciousness (Ayer, 1963). But how exactly can a normal present-day person, a mere human, have direct knowledge of what is happening in the mind of another? Is this genuinely possible, or is it one of those scenarios that merely seems possible but actually is not - such as perpetual motion or space travel faster than the speed of light?

Another sort of objection to the idea of linking brains is doubtless on the minds of many readers at this point. In order for one person to experience the consciousness of another, there needs to be a distinction in our minds between the experiencer and the object of experience. But this distinction cannot be made: there is no such thing as an independent experiencer, so the objection goes. The problem is that it seems that both the state and our experience of it are combined in a simple, unbreakable, monolithic unit, as in Searle's view. It is also widely believed that there is no separate experiencer, no self. None has ever been found by science, and philosophers as far back as David Hume (1739) dispute that introspection reveals one.

Contrary to all these lines of thought, I believe that a clear separation can be made in the mind and brain between our conscious mental representations and the other processes that both interact with these representations within consciousness and give rise to our sense of self, the sense of an experiencer. According to this hypothesis, what we call introspection consists of causal interaction between our mental representations and these other processes that produce a sense of self. These two components are, I will suggest, generated by different brain processes and hence the separation needed to allow us to explore the possibility of one person experiencing, or introspecting, the conscious mental representations of another person exists.

It may be that what the mysterians and the subjectivists have done is not show that the problem of consciousness is insoluble but, rather, that it is insoluble given the assumption of privacy. It is time to consider the possibility that the failure of the existing views to solve the problem amounts to an argument against them, in the same way that scientific theories that go too long without being able to solve crucial problems become suspect. In this section, I will begin construction of an alternative conception; first, by describing some of the evidence that real and effective brain processes underlie our sense of self and that these processes are separate from the brain processes that produce, prepare, and embody conscious mental representations. The many questions raised by such a view require detailed explaining and defending. They include issues in psychology and neuroscience, as well as philosophical issues treated in the philosophical subfields of metaphysics and epistemology, as well as philosophy of language (Hirstein, 2008). Here, I will focus on the basic components of the view and marshal some of the primary pieces of evidence for them, drawing primarily on findings in neuroscience, especially from cognitive neuropsychology. I will describe a hypothesis according to which higher cognition in the human brain is characterized by these self-like processes causally interacting with perceptual (or mnemonic) representations, which have been carefully modified and prepared to interact with them. That will be followed by a list of several different ways in which the perceptual representations we are aware of are prepared and modified prior to our awareness of them. Second, I will discuss the phenomenon of binding, a type of brain event that allows several different processing streams to unify to form a single, coherent conscious state. Finally, using this information, I will explain how mindmelding, the direct experience by one person of another's conscious representations, is in fact possible.

In this alternative view, the existence of our conscious states can be separated from our knowledge of them. If Searle and the other believers in subjectivity and privacy are correct, mindmelding is metaphysically impossible, since it requires a separation between the object and our knowledge of it, and that can never be achieved – it would be like trying to split an ultimate particle, a true atom. The alternative suggested here is that, contrary to the views of Searle and others, no conscious state has its existence necessarily tied to our knowledge of it. Thus, there is no need to invent a new metaphysical category in order to describe and explain consciousness. Consciousness is surely necessary in order for a person to know about, or be aware of, something; but contrary to Searle's view, consciousness alone is not sufficient. The proper causal relations between our conscious representations of that thing and our executive processes must also exist. The primary causal relation in this regard is what we normally call “attention.” Here is an example of what I mean: In the visual modality, one's focus of attention moves around within the visual field. It is typically located where the eyes are focused, but it need not be - it is possible to visually attend to something without looking at it. We *can* be aware of the periphery outside the visual focus, but we normally are not, in the same way that we are not aware of feeling the shoes on our feet until we attend to it. There are now several different experiments that show that the information in the non-attended portions of the visual field cannot be reported by subjects (Rensink, O'Regan and Clark, 2003). The unattended portions of the visual field are still part of a conscious brain state, it is just that it is not a part that we are currently aware of. This difference is captured by our everyday distinction between “consciousness” and “consciousness of.” Something can be conscious in our minds without our being conscious of it.

There is wide agreement among brain scientists that higher-level mental processes, including conscious representation and thought, take place in the cortex, the folded grey outer layer of the brain. It is now a truism that perceptual processes are located primarily in the back of the cortex, while motor, or at least action-oriented processes, are located in its frontal regions. Unlike simpler species, our perceptions and the actions based on them can be widely separated in time. We think about things, mull the situation over, contemplate, deliberate, correct our ideas, and rethink matters. We make decisions, some of which take seconds, while others can take years. There are two basic participants in this decision-making process. First, since correct representations of the world are crucial to good decisions, one participant in decision-making is the huge set of mental representations contained in our brains. But some other brain processes need to employ these representations to actually make a decision and direct actions out into the world in an effective way. These latter processes also need to mix in the effects of emotions, motivations, and memories during the planning and decision-making process. Neuroscientists call these other brain processes *executive processes* (Stuss *et al*., 2002). They are, I will contend, one of a set of phenomena that produce our sense of self, including the sense that we are in charge of our thoughts and actions.

### Executive Processes and Sense of Self

How many minutes of the day do you spend actually thinking? If it is a normal day and you are a normal person and not, say, the leader of a country or a large corporation, probably not many. We often go through an entire day performing actions we have performed dozens or even hundreds or thousands of times before, expending very little conscious mental effort. But when something important is at stake, or something unexpected or negative happens, we need to break out of our routines and solve problems or decide what to do. Neuropsychologists have found that executive processes are required to actively stop routine actions and initiate decision-making or problem-solving processes (Gazzaniga, Ivry and Mangun, 1998). Otherwise, a phenomenon known as *perseveration* takes place: We keep doing the same thing even when we can see that it is not working. Our notion that there is something like a self at work in our minds is produced partly by executive processes that manipulate representations, eliciting them, monitoring them, checking them, correcting them, using them to guide actions, or stopping them from leading to actions. These processes create an active presence in the conscious mind, a sense that there is something there interacting with our representations. The following situations require the intervention of executive processes: when planning or decision making is required; when there are no effective learned input-output links; when a habitual response must be inhibited; when an error must be corrected; when the situation is dangerous; when we need to switch between two or more tasks; or when we need to recall something.

A great deal of what we normally call thinking, deciding, planning, and remembering is accomplished primarily by the brain's executive processes. One introspectively accessible measure of the amount of executive activity is our sense of mental effort. Increased mental effort correlates with increased usage of oxygen by executive areas, which is detectable by brain imaging. Most executive processes reside in the prefrontal lobes, including the dorsolateral frontal lobes, on the side of the brain, the ventrolateral frontal lobes below them, and the orbitofrontal lobes, located just above the eye sockets (Fuster, 2002; Rolls, 1999). This sense of an active presence in the mind, accompanied by a sense of mental effort are, I suggest, important in the etiology of our notion of the self. Not the ongoing sense of self over time, accomplished in large part by our autobiographical memories (Tulving, 1993), but a sense of self in the moment. According to our everyday way of thinking of it, the self is a mental entity that performs various functions: it confronts perceptual data as it enters the mind; it makes decisions; it initiates voluntary actions “(*self*” and *will* are intimately connected);” and it inhibits ill-formed intentions from being acted upon. All of these mental tasks are accomplished by executive functions.

Damage to one prefrontal executive area, the anterior cingulate, can cause a profound inability to act, called akinetic mutism or vigilant coma (Nemeth, Hegedus and Molnar, 1988). The patient appears awake and alert but will not respond to stimuli or to requests or commands from doctors. Patients who recover report that they perceived the stimuli and understood the requests but simply had no impetus to act - a disorder of the will. It is also of interest that we tend to use the word “I” when describing what the executive processes do. We say, I am aware of x; I recalled that x; I stopped myself from doing x; I thought about x; I analyzed the idea that x; I decided to x; I plan to x; and I intend to x - all of which ultimately refer (in some way) to the operation of an executive function. In contrast, impulses, thoughts, and images that pop up into consciousness are the data that the executive processes operate on. When such mental events occur that are not the result of executive processes but which causally affect the executive processes, we speak in a passive mode: My attention was drawn to x; an image of x flashed in my mind; it occurred to me that x; I was distracted by x. Introspection in this conception is analogous to perception. Introspection involves something like a perceiver and something like an object of perception. What we think of as introspection occurs when certain executive processes participate in certain types of causal relations with conscious representations. The executive processes are analogous to the perceiver and the representations are analogous to the objects of perception.

**Figure 1:**
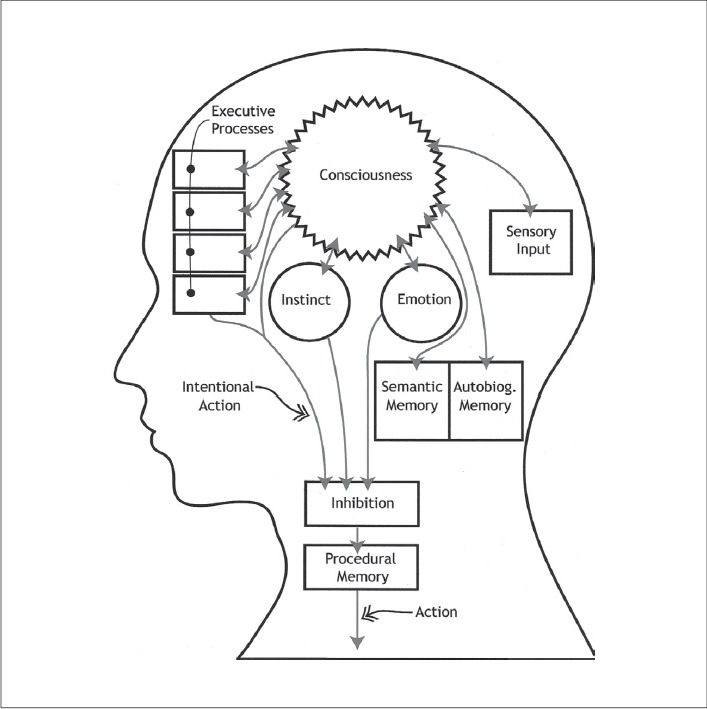
Functional diagram of the brain. (This schematic of the brain's functions embodies the idea that the executive processes in the prefrontal lobes are separate from our conscious representations in the temporal and parietal lobes. The executive processes interact with our conscious representations, as well as neural systems embodying our emotions and instincts, to initiate (or if necessary inhibit) actions. Diagram by Xavier Arko).

According to several theorists, the prefrontal cortex does not contain our conscious mental representations (see, e.g., Miller and Cohen, 2001; Petrides, Alivisatos and Frey 2002). They reside in more posterior regions, in the temporal and parietal lobes. The prefrontal lobes contain the executive processes that monitor and manipulate these representations (Stuss *et al*., 2002). Thus the function of the prefrontal cortex [PFC] and its executive processes is “modulatory rather than transmissive. That is, the pathway from input to output does not ‘run through’ the PFC. Instead, the PFC guides activity flow along task-relevant pathways in more posterior and/or subcortical circuits” (Miller and Cohen, 2001). More specifically, the dorsolateral PFC is responsible for “the monitoring of multiple events within working memory, regardless of the nature of the stimulus…rather than the maintenance of the stimuli *per se*,” which occurs in posterior, temporal and parietal areas (Petrides, Alivisatos and Frey, 2002). Claims such as these support the idea that it is possible in principle to separate the executive processes in the prefrontal lobes from the representations they operate on, in the temporal and parietal lobes [Figure 1].

The late Francis Crick and his collaborator Christof Koch called the process in which information from the different sensory modalities is bound into a unified multimodal representation a process of producing an “executive summary” (Koch, 2004) - an the analogy of the way that employees will summarize the important information and eliminate potentially distracting details in any document they give to their supervisors. Vision, for instance, involves an interaction between our brains’ clearest “interpretation” of its current visual information and the frontal areas that operate on this interpretation to produce intelligent action. According to Crick and Koch:

*The biological usefulness of visual awareness…is to produce the best current interpretation of the visual scene, in the light of past experience…and to make it available, for a sufficient time, to the parts of the brain that contemplate, plan, and execute voluntary motor outputs… (Crick and Koch, 1995)*.

*A single, compact representation of what is out there is presented, for a sufficient time, to the parts of the brain that can choose among different plans of action (Koch, 2004)*.

I suspect one reason why Crick and Koch are comfortable using verbs such as “contemplate,” “plan,” and “choose” in describing executive activity is that they know that this way of speaking is easily defended against the old “homunculus objection,” the charge that they are endowing some part of the brain with all the abilities of a full human mind, which would make the explanation circular (Dennett, 1978). The idea that the executive processes need their representations prepared for them implies that they are quite limited, unlike the entire person, who is capable of dealing with ambiguities and vagueness. The executive processes are also severely limited in that they each accomplish only one of the many mental functions of the human mind, nothing at all like a homunculus, which presumably can do whatever the full person can do.

## The Brain's Higher-Level Architecture

A great deal of processing by the brain's perceptual areas occurs before we are aware of events in our surrounding environment. Perception is a multistage process in which incoming energy in several different forms - chemical, electrical, and mechanical - must be transduced into certain types of electrical impulses, processed, and structured so that the executive processes can causally interact with the resulting representations. Our initial perception of the world is complicated and multi-leveled so that incoming information can be put into exactly the form that the executive processes work best and commit the fewest errors with (Ramachandran and Hirstein, 1997). According to this hypothesis, at some point long ago in its evolutionary history, the human brain developed a special kind of engineering approach to solving the problems of existence in a fiercely competitive environment: an architecture in which executive processes operate on highly processed representations.

This hypothesis suggests that rather than evolving single-level, highly detailed representations, we evolved stratified representations, with each level at a relatively low resolution. For instance, the visual field we experience is a combination of several levels, each of which is constructed by a separate brain process. After the levels are constructed, they are bound together into a coherent and unified representation of our surrounding environment. The colours of objects are bound with their shapes, and these are bound with information about which objects are in front of which. At another level, objects are identified by being associated with concepts. This construction process is for the benefit of the executive processes. For instance, the addition of a level in which colour is added to visual representations makes the work of the executive systems much easier. Colour differences make more salient those stimuli that would be extremely difficult to detect with black and white vision, even with a higher resolution. Colours parse the visual field in a clear and consistent way, making the projection of actions into that part of the environment easier and more effective.

According to this hypothesis, the layering of levels within representations is only one of many techniques the brain has for preparing representations to interact with executive processes. Others include:

*Filling in*: The clearest case of preparation is seen in the filling in of the blind spot. In the last 15 years several pieces of evidence have emerged to support the claim that the brain's visual cortices have processes that fill in the blind spot based on what is being perceived in the surrounding area (Ramachandran and Churchland, 1994; Pessoa and De Weerd, 2003).

*Colour constancy*: We see the colours of objects as remaining the same under different lighting conditions and at different viewing angles, whereas in actuality there are subtle changes in these different conditions.

*Colour uniformity*: We perceive surfaces as being of a uniform single colour but, typically, there are variations in the colour over a surface. Again, as with colour constancy, this gives the impression of preparation and simplification of the visual scene.

*Border sharpening*: The perceptual systems parse their data into chunks and then artificially sharpen the boundaries of those chunks. In vision, the borders of objects are made sharper and continued into areas where no border is visible (even outside the blind spot) as many of the classical Gestalt phenomena show. In the auditory realm, when we hear someone speak, the divisions between words are sharpened.

Thus the brain extensively edits and prepares its representations before they interact with prefrontal executive processes. Perhaps because what the executive processes accomplish is a high-level, highly effective form of cognition that is quite rare in the animal kingdom, they need help from other parts of the brain to tailor and adapt their products to compensate for their limitations.

### Binding

Now that I have outlined my case for the claim that a coherent separation can be made between conscious representations and the executive processes that operate on them, I need to bring them back together into a single “interpersonal” conscious state. In our normal conscious lives, we experience unified mental events in which executive processes interact with representations. The brain's act of joining different states and processes into a single conscious state has come to be called *binding* (Crick and Koch, 1990). Scientists first began to understand the brain by tracing the input from the sense organs to what are called unimodal cortical areas - areas devoted to a single sensory modality. They found visual areas, auditory areas, olfactory areas, areas devoted to processing information about the body (called somatosensory areas), and areas devoted to processing different tastes (called gustatory areas). As they traced these causal chains inward, they found that processing in each modality progresses through several different levels (see Zeki, 1993, for an example in the visual modality). They saw that, once this information has been fully processed, the unimodal areas converge on several different multimodal areas (Macaluso and Driver, 2005). But the scientists then realized they had a difficult question on their hands: How do the multimodal areas combine their inputs into the seamless unified experience we have? This has become known as the “binding problem” (Crick and Koch, 1990).

There are apparently several different levels of binding in the brain. It occurs not only across modalities but also within modalities. For instance, as I have noted, certain areas of the brain produce the object shapes we see while other, connected areas produce the colours of these objects; but in our conscious perception the colours and shapes are combined. Several different levels of binding are needed to produce a full conscious mental state:

1. Binding of information from many sensory neurons into object features
2. Binding of features into unimodal representations of objects
3. Binding of different modalities, e.g., the sound and movement made by a single object
4. Binding of multimodal objects into a representation of a full surrounding environment
5. Binding of representations, emotions, and executive processes, etc., into full mental events

Research into how binding is accomplished is in its infancy (see the essays in Cleermans, 2003). Most theorists of binding posit electrical oscillations generated by nerve cells that synchronize the activities of different cortical areas through phase-locking, i.e., all of the bound areas begin resonating together, typically at around 40 Hertz (Singer, 1997). We do not yet know, however, whether these different levels of binding are achieved by one process or by several different ones. The conscious mental events we experience are not simply unified, highly prepared, multimodal conscious representations; they are events in which things are done with representations. This implies that the executive processes are also bound with the sensory information to form larger mental events, as in the fifth type of binding mentioned above.

## White Matter Fiber Tracts

This separation, between those brain processes that embody our conscious representations and those that manipulate them and produce a sense of self, is crucial to the possibility of one person experiencing the conscious representations of another. If conscious perceptual representations are located toward the back of the brain - specifically, in the temporal and parietal lobes - and sense of self is generated by processes located toward the front - in the prefrontal lobes - what if we imagine connecting person A's temporal lobes to person B's prefrontal lobes? Could this be done in a way that would produce a coherent conscious state for B? Could this produce a case where one person, B, has direct access to another person's, A's, perceptual representations? I think that this is a real possibility. Making the connection should not be monumentally difficult since those areas are already connected by fiber tracts. These bundles have close connections to consciousness, in that whatever affects them has immediate effects on consciousness. Whether mindmelding of this sort should ever actually be attempted on humans, I see as a very sensitive ethical issue; I address that in the final section.

**Figure 2:**
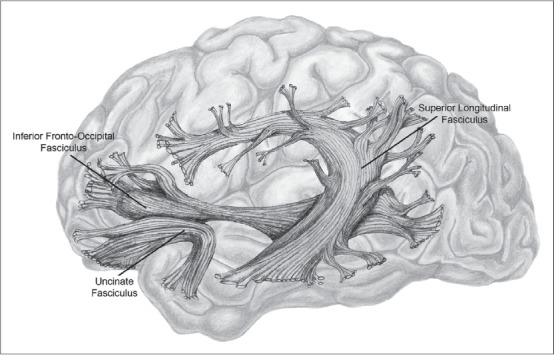
White matter fiber tracts. (These are the three major tracts connecting the temporal and parietal lobes with the prefrontal cortex. Diagram by Katie Reinecke, after Kier *et al*., 2004.)

Nature has provided the perfect structure to allow us to perform mindmelding experiments. The temporal and parietal lobes are extensively interconnected with the executive processes in the prefrontal lobes by several different white matter fiber tracts, called association fibers (Schmahmann and Pandya, 2006). These bundles of fibers, also known as fasciculi, are made up of millions of connecting fibers, which are axons protected by an insulating myelin sheath. “Gray matter areas operate in concert with each other in the mediation of [higher functions], and white matter forms the connecting tissues that link these areas into coherent neural assemblies” (Filley, 2001). The causal flow within these tracts is little like the orderly movement of electrical impulses along computer data transmission lines, those ribbons of gray wires that connect the different parts of the typical desktop computer. For one thing, there is causal flow in both directions (Filley, 2001). The primary fiber tracts connecting the temporal and parietal lobes with the prefrontal cortex are the superior longitudinal fasciculus, the inferior frontooccipital fasciculus, and the uncinate fasciculus (Ungerleider, Gaffan and Pelak, 1989) [Figure 2].

There is evidence that these fiber bundles have important connections to consciousness, in that damage to them has immediate effects on consciousness. According to Kier *et al*. (2004), the uncinate fasciculus and the inferior occipitofrontal fasciculus play a role in producing hallucinations when their connected areas are damaged. Hubl *et al*. (2004) found that schizophrenics who experienced auditory hallucinations had marked differences in their association fibers (in the arcuate fasciculus, another fiber bundle connecting the temporal and prefrontal lobes), which led to “disrupted frontotemporal processing.” Kier *et al*. (2004) also note that, “patients who undergo anterior temporal lobectomy have object- and action-naming deficits resulting from the disruption of frontotemporal connections mediated by the uncinate fasciculus” (Kier *et al*., 2004). Koch (2004) says that some projection neurons from inferior temporal areas to the principal sulcus in the prefrontal cortex may form part of what he and others call the neural correlates of consciousness.

## Mindmelding

**Figure 3:**
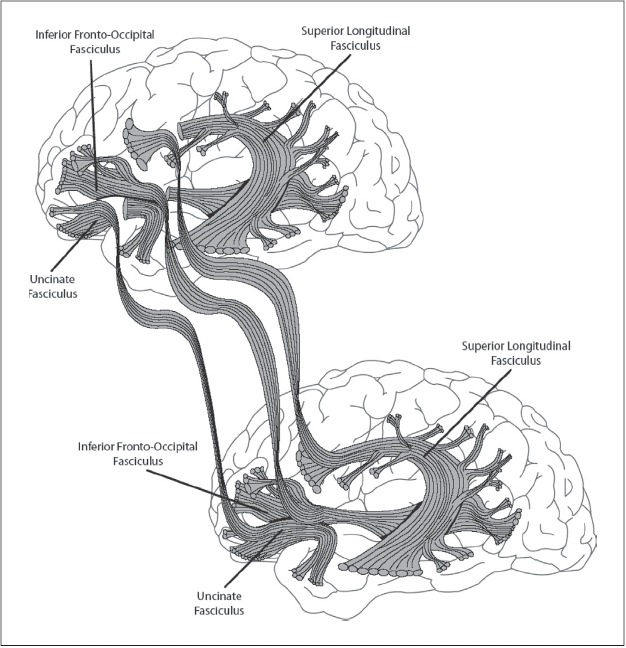
Mindmelding experiment. (The owner of the brain on top can experience the conscious representations of the owner of the brain on the bottom. What the person on top experiences cannot be his own conscious perceptual representations, which reside in his temporal and parietal lobes, since the connections to those have been severed. Diagram by Katie Reinecke.)

Imagine two normal people standing side by side. Call them A and B. With a bit of imagination we can create different mixed nervous systems, made from parts of A's and B's nervous systems, as they continue to function. We might imagine shunting the input running up A's optic nerves to B's visual system, for instance. Then B would literally see things through A's eyes. As a first thought experiment aimed at producing mindmelding, we might try connecting A's temporal lobes to B's prefrontal lobes. We might imagine grasping the temporal lobes and pulling them out away from the rest of the brain. Imagine that all of the connections between the temporal lobes and the brain are able to stretch, so that as we pull the temporal lobe away we begin to see all the connections. What if we then branched all of the fiber tracts in A's brain and ran a connection to B's brain [Figure 3]? We connect all these fiber bundles topographically to the other brain, matching each fiber to its nearest topographic equivalent in the other bundle. We would also need to make all of the other physical connections required for binding to occur. This would involve many of the important specific and nonspecific connections between the thalamus and the cortex (Jones, 2002). Could this produce a unified conscious state?

A tuning period may be required in order for the two brains to work effectively, but the central nervous system is very good at this sort of tuning and adjustment, as the abundance of recent findings on the brain's plasticity, especially in response to injury, affirms (Buonomano and Merzenich, 1998; Ramachandran and Hirstein, 1998).

One thing it means to claim that B experiences the conscious states of A is that B might truly say after the procedure, “I just experienced A's conscious visual states, and what he calls ‘red’ is actually green!” Mindmelding would be a strange and possibly frightening experience for B, because he would be aware that the conscious representations are in some important sense not his, but he would nevertheless experience them in the intimate way normally reserved for one's own conscious representations. As we have described it, the conscious states that B apprehends would be modified by the executive processes of A, and this would give B an odd feeling of lack of control over them. In the early days, it may be necessary to simply block the causal influences coming from either A or B's frontal regions, to avoid having two different sets of executive processes attempting to manipulate the same representation. Or we might imagine putting in place the connections that would allow B's executive processes to manipulate A's conscious representations, then perhaps A and B could take turns having control over A's representations. If B's executive processes are able to manipulate the representations of A, this is conceivably a case of B introspecting the mind of A.

It is important to be clear that mindmelding does not involve one person having access to a *copy* of another person's conscious representations. Even if we were satisfied with the fidelity of a copy, experiencing it would still be an indirect way to know about the mental states of another. If there were ever disagreement between A and B about what was being experienced, we would have to give much greater weight to what A said, since A is in possession of the original. In mindmelding, B is in contact with A's conscious representations themselves. A and B are directly aware of the same states and processes, and in the same way. Person B apparently cannot coherently experience A's *entire* conscious state, i.e., her conscious representations plus her executive processes, but this is not because A's conscious states belong to some special metaphysical category, as in the subjectivist approach. Rather, it is a product of the way we experience the world and the way in which we identify with what the executive processes do. I cannot experience your entire conscious state without being you. But if I were you, this would not be a case of my experiencing your consciousness, but rather just another case of you experiencing your consciousness. It would not be me knowing you but, rather, me becoming you. There simply is no “room” for two people in anything resembling a normal, full conscious state. (See De Vignemont, 2004, for a description of other types of states in which two people are “co-conscious”). My goal here is simply to show that privacy, i.e., the claim that one person's conscious mental life is completely closed to others, is false, by showing that portions of one person's conscious state can be experienced by another. One cause of confusion here is that the phrase “what it's like to be x” (Nagel, 1974) is ambiguous between a reading in which one experiences some portion of x's consciousness and a reading in which one must experience the totality of x's consciousness. But the subjectivists are quite clear that *no* portion of x's conscious state can be experienced by another and it is this claim that I am addressing.

Let me end this section by providing brief answers to several pertinent questions. Has such an experiment ever been done on humans or animals? Not to my knowledge. It would be useful to do the necessary experiments on animals in order to attempt human mindmelding, provided a therapeutic use is evident; but this would not be helpful with the metaphysical/epistemic problem, since we then have the question of trying to figure out what the animal is experiencing. Is it possible to do such an experiment in the foreseeable future? I think we are close to assembling the needed expertise and technology. Recent work on repairing the spinal cord might also prove useful here (Iwata *et al*., 2006). Has any preliminary work already done? There are several projects underway to connect human neural tissue with computer processors (Clark, 2003), including experiments in which chimpanzees are trained to move a computer cursor or a robotic arm merely by thinking (Carmena *et al*., 2003). Is this idea not merely science fiction? Mindmelding at the moment is a “thought experiment’; my primary point is that such an experiment is possible and conceivably could produce mindmelding, because so many philosophers and scientists believe that this is impossible.

## Concluding Remarks

The possibility of invading a person's normally private mental life raises serious ethical questions, questions similar to those recently asked with regard to the attempts to devise a more accurate lie-detection device using brain-imaging techniques (Greely and Illes, 2007). Some will argue that this is an area into which we simply should not go, a possibility that should not be explored. Some mysteries should be left mysteries, they argue. This is obviously not my position. My position is that it is always better to know the truth, whatever it is. Wherever mystery exists, there are people who will attempt to use our lack of knowledge for their own gain. What happens to us after death, for instance, is perhaps the greatest mystery of all, and there are countless con men who claim to be able to tell us exactly what happens or even to communicate with our dead relatives. Closer to home, there are people who claim to be able to read thoughts. These people play on our hopes and fears, drawing their power from the mystery.

Philosophy is more disinterested than medicine, but the preoccupations, even biases, of medicine are not unwelcome. The main purpose of understanding the human mind is to better treat its diseases and derangements. Seeing the mind-body problem as a medical problem thus provides a context in which the ethical issues involved can be understood, including severe violations of mental privacy. We have always made a sharp distinction between privacy in everyday situations and in medical contexts. Seeing someone naked, for example, is a great violation of privacy, but is frequently necessary in order for the doctor to do her work; modesty and privacy should not interfere with diagnosis and monitoring for medical purposes. Diagnosing illness and monitoring the effectiveness of treatments are essential in solving medical problems. Diagnosing tinnitus, for instance, involves asking the patient about the exact quality of the sound he is aware of. With mindmelding, the doctor would have the option of actually hearing the sound itself, rather than relying on our limited ability to describe sounds. The great mysteries surrounding the question of what the mental lives of certain patients are like, for instance the autistic person's or the schizophrenic's, might be approached with mindmelding.

### Take Home Message

Consciousness is not essentially private. Conscious states are physical states like any other. We can show that a separation can be made between the existence of conscious states and our awareness of them by connecting two brains so that one person is aware of the conscious states of another. But the connection must be in the correct places, and our understanding of the metaphysical and epistemic natures of the conscious states involved must also be modified, if we are to correctly conceptualize the phenomena.

## Questions That This Paper Raises

1. Can someone experience another's sense of self, or would the first person lose his own sense of identity in attempting to do so?
2. How should we deal with the ethical problems raised by breeching the wall of privacy?
3. Do dolphins and other large-brained mammals also possess a system of executive processes?
4. Why do our minds present themselves to us as non-material?
5. Why has the assumption that our minds are essentially private gone unquestioned for so long?

## About the Author

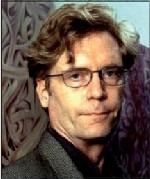



William Hirstein is Chair of the Philosophy Department at Elmhurst College, in Elmhurst, Illinois, USA He received his PhD from the University of California, Davis, in 1994. His graduate and postdoctoral studies were conducted under the supervision of John Searle, V. S. Ramachandran, and Patricia Churchland. He is the author of several books, including On the Churchlands (Wadsworth, 2004), and Brain Fiction: Self-Deception and the Riddle of Confabulation (MIT, 2005). His other interests include autism, sociopathy, brain laterality, and the misidentification syndromes.
